# Predictive Value of Liver Enzymes and Inflammatory Biomarkers for the Severity of Liver Fibrosis Stage in HIV/HCV Co-Infected Patients

**DOI:** 10.1371/journal.pone.0059205

**Published:** 2013-03-19

**Authors:** Charlotte Charpentier, Karen Champenois, Anne Gervais, Roland Landman, Véronique Joly, Sylvie Le Gac, Lucile Larrouy, Florence Damond, Françoise Brun-Vézinet, Diane Descamps, Yazdan Yazdanpanah

**Affiliations:** 1 Laboratoire de Virologie, Assistance Publique-Hôpitaux de Paris (AP-HP), Groupe Hospitalier Bichat-Claude Bernard, HUPNVS, Université Paris Diderot, Paris 7, PRES Sorbonne Paris Cité, EA4409, Paris, France; 2 Inserm ATIP-Avenir: "Modélisation, Aide à la Décision, et Coût-Efficacité en Maladies Infectieuses", Lille, France; 3 Service des Maladies Infectieuses et Tropicales, AP-HP, Groupe Hospitalier Bichat-Claude Bernard, HUPNVS, Université Paris Diderot, Paris 7, PRES Sorbonne Paris Cité, EA4409, Paris, France; University of Pittsburgh Center for Vaccine Research, United States of America

## Abstract

**Objective:**

The aim of our study was to assess a possible association between plasma inflammatory biomarkers (CRP, IL-6, soluble CD14) and the extent of fibrosis or cirrhosis using a FibroScan® in HIV/HCV co-infected patients.

**Methods:**

This cross-sectional study assessed 60 HIV/HCV co-infected patients who had paired plasma samples and FibroScan® values available. All included patients were controlled for HIV infection (HIV-1 RNA <50 copies/mL) and had detectable HCV RNA levels. Levels of three biomarkers were measured in all samples using commercial ELISA kits. Multivariate logistic regression models identified factors associated with the METAVIR stages of fibrosis (F0–F2 *vs.* F3–F4).

**Results:**

In univariate logistic regression analyses, in addition to sCD14 (odds ratio [OR] = 3.23, 95% confidence interval [95%CI] = 1.30–7.97, *P = *0.01), aspartate aminotransferase (AST), alanine aminotransferase, platelet counts, and CD4 cell counts were associated with the stage of liver fibrosis and, thus, were introduced into the model. However, only AST (OR = 1.06, 95%CI = 1.02–1.10, *P* = 0.0009) was independently associated with F3–F4 stage liver fibrosis.

**Conclusions:**

In our study of HIV/HCV co-infected patients, sCD14 plasma level, a biomarker of monocyte activation, was not independently associated with the F3–F4 stage of liver fibrosis. We hypothesize that the higher levels of inflammation markers observed in HIV/HCV co-infected patients, compared to HCV mono-infected patients, prevent this association being observed within this population.

## Introduction

Microbial translocation has been described as associated with chronic inflammation in both HIV-infected and hepatitis (HBV or HCV)-infected patients [1]–[4]. The mechanism of microbial translocation is positively correlated with the level of the bacterial product, lipopolysaccharide, and is indirectly reflected by the presence of soluble CD14 (sCD14), a plasma biomarker of monocyte/macrophage activation.

In HIV-infected patients, several studies reported that high sCD14 plasma levels predict disease progression and are positively correlated with all-cause mortality [2], [3]. Higher levels of the inflammatory biomarkers, C-reactive protein (CRP) and interleukine-6 (IL-6), have been also previously associated with an increased risk of mortality or opportunistic diseases in large randomized clinical trials [5], [6]. Similarly, the role of sCD14 has been reported in patients infected with HBV or HCV, as well as an association between high sCD14 plasma levels and cirrhosis; thus, the presence of sCD14 can predict progression to end-stage liver disease [4]. However, there are scarce data on the role and predictive value in the development of liver fibrosis of inflammatory biomarkers as high-sensitivity CRP (hsCRP) and IL-6, or of a monocyte/macrophage activation marker, such as sCD14.

The aim of our study was to assess the possible associations between plasma inflammatory biomarkers and the extent of liver fibrosis or cirrhosis in HIV/HCV co-infected patients.

## Patients and Methods

This cross-sectional study included co-infected HIV/HCV patients from the Infectious Diseases Unit of Bichat Hospital (Paris, France). All enrolled patients were receiving an antiretroviral-based therapy and were virologically suppressed for HIV replication (HIV-1 RNA <50 copies/mL), they had detectable HCV RNA levels, and paired plasma samples and FibroScan® values were available. Plasma samples were obtained between 2009 and 2011, and stored at −80°C in Bichat Hospital virology lab.

This study's protocol was approved by an institutional ethics review board (Saint-Germain en Laye, France), and all patients provided their written informed consent.

Levels of three biomarkers were measured in all samples. hsCRP (human C-reactive protein [CRP] Quantikine ELISA Kit, R&D Systems, MN, USA), hsIL-6 (human IL-6 Quantikine High Sensitivity ELISA Kit, R&D Systems), and sCD14 (human sCD14 Quantikine ELISA Kit, R&D Systems) were measured using commercial ELISA assays. Lower levels of detection were 0.010 ng/mL, 0.039 pg/mL, and 0.125 µg/mL for hsCRP, IL-6, and sCD14, respectively.

Sociodemographic characteristics and clinical, virological, and biological data from patients were assessed using descriptive statistical methods. We assessed the correlation between inflammatory biomarkers and FibroScan® values as continuous variables using Spearman’s rank test. We also used a logistic regression model, as a dichotomous variable, to determine if there was an association between the level of inflammatory biomarkers and METAVIR stage of liver fibrosis (F0–F2 *vs.* F3–F4). Then we adjusted the model for those factors found, in univariate analyses, to be associated with METAVIR fibrosis stage (F0–F2 *vs.* F3–F4). We applied a forward-selection procedure for those variables with a univariate *P*-value of ≤0.20, and we retained a significance threshold of *P = *0.05 to determine which factors were independently associated with the stage of liver fibrosis. Analyses were conducted with SAS software, version 9.2 (SAS Institute, Cary, NC).

## Results

The characteristics of the 60 included patients are shown in Table 1. Their median age was 50 years (interquartile range [IQR] = 46–53) and 75% were men. Among all patients, 28 (47%) were receiving a protease inhibitor-based regimen, 15 (25%) a non nucleoside reverse transcriptase inhibitor-based regimen, and 17 (28%) a different antiretroviral-based regimen. Most patients (*n* = 36, 60%) were infected with HCV genotype 1, three (5%) with HCV genotype 2, six (10%) with HCV genotype 3, and fifteen (25%) with HCV genotype 4. Seventeen patients (28%) displayed METAVIR F3- or F4-stage liver fibrosis (FibroScan® value ≥10.5 kPa). The median time since diagnosis of HIV infection was 19 years (IQR = 11–23). The median CD4 cell count at the time of the study and nadir CD4 cell count were 562/mm^3^ (IQR = 291–771) and 177/mm^3^ (IQR = 70–255), respectively.

**Table 1:** Characteristics of HIV/HCV co-infected patients (*n* = 60).

Characteristics	*n* = 60
Men *n* (%)	45 (75)
Age (years)	50 (46–53)
HCV RNA (log_10_ IU/mL)	6.46 (6.06–6.70)
HCV genotype 1 *n* (%)	36 (60)
AST (U/L)	48 (37–65)
ALT (U/L)	57 (42–83)
GGT (IU/L)	78 (44–133)
Creatinine (µM/L)	77 (64–88)
Platelet count (/µL)	174 (129–217)
CD4 cell count (/mm^3^)	562 (291–771)
Nadir CD4 (/mm^3^)	177 (70–255)
Duration since diagnosis of HIV infection (years)	19 (11–23)
CD4/CD8 ratio	0.69 (0.41–0.98)

Continuous variables were expressed as medians and interquartile ranges (IQR), and categorical variables were expressed as numbers and percentages.

ALT: alanine aminotransferase; AST: aspartate aminotransferase; GGT: gamma glutamyl transpeptidase.

**Figure 1:**
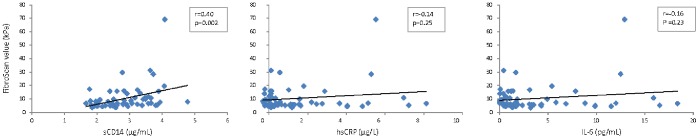
Correlation between FibroScan® values and inflammatory biomarkers (Spearman rank’s test).

In the 60 patients, median sCD14, hsCRP, and IL-6 levels were 2.79 µg/mL (IQR = 2.39–3.46), 0.67 µg/L (IQR = 0.19–2.07), and 1.73 pg/mL (IQR = 0.47–4.91), respectively. The FibroScan® values correlated with sCD14 plasma levels (r = 0.40, *P = *0.002), but not with IL-6 (r = 0.16, *P* = 0.23) or hsCRP-plasma levels (r = 0.14, *P* = 0.25) (Figure 1). In the logistic regression, which included only inflammatory markers, sCD14 was associated with the F3–F4 stage of liver fibrosis (odds ratio [OR] = 3.35, 95% confidence interval [95%CI] = 1.31–8.53, *P = *0.01).

In the univariate logistic regression analyses, in addition to sCD14 and ALT, aspartate aminotransferase (AST) (*P = *0.0009), platelet counts (*P = *0.0055), and CD4 cell counts (*P* = 0.05) were associated with the stage of liver fibrosis and so were introduced into the model (Table 2). However, only AST (OR = 1.06, 95%CI = 1.02–1.10, *P* = 0.0009) was independently associated with the F3–F4 stage of liver fibrosis (Table 2).

**Table 2:** Factors associated with the METAVIR liver fibrosis stage.

	F0–F1 *n* = 43	F3–F4 *n = *17	OR	[95% CI]	*P*-value	aOR	[95% CI]	*P*-value
AST (U/L)	42 [34–56]	71 [56–107]	1.06	[1.02–1.10]	0.0009	1.06	[1.02–1.10]	0.0009
ALT (U/L)	52 [36–67]	97 [59–135]	1.03	[1.01–1.05]	0.0017	–		
Platelet count (/µL)	183 [155–231]	129 [76–194]	1.00	[1.00–0.01]	0.0055	–		
sCD14 (µg/mL)	2.57 [2.15–3.07]	3.33 [2.82–3.66]	3.23	[1.30–7.97]	0.01	–		
CD4 cell count(/mm^3^)	630 [453–788]	455 [248–567]	1.00	[0.99–1.00]	0.05	–		
Sex, men, n (%)	30 (70)	15 (88)	3.25	[0.64–16.30]	0.15	–		

Continuous variables were expressed as median and interquartile range (IQR).

ALT: alanine aminotransferase; aOR: adjusted odds ratio; AST: aspartate aminotransferase; OR [CI95%]: odds ratio and 95% confidence interval.

Similar results were obtained in a multivariate regression model, which evaluated the association between inflammatory biomarkers and FibroScan® values, adjusted on other variables (data not shown).

## Discussion

In this study, based on 60 HIV/HCV co-infected patients, we found high levels of inflammatory biomarkers. However, sCD14 plasma level, the biomarker of monocyte activation, was not found to be independently associated with F3–F4 stage liver fibrosis.

A main limitation of our study was its limited sample size, which meant there was lack of statistical power; however, we assumed that the risk of selection bias was negligible. Furthermore, few studies have reported on the role of inflammatory biomarkers in HIV/HCV co-infection and, as yet, no large studies have been conducted.

In our study, the median sCD14 plasma level was 2.79 µg/mL (IQR = 2.39–3.46), which is a similar level to that observed in the study of Marchetti et al.: they assessed inflammatory biomarkers in HIV/HCV co-infected patients and found that median sCD14 plasma levels ranged from 2.79 to 3.09 µg/mL and was linked to the severity of the liver disease [3]. In a study by Sandler et al., which assessed the inflammatory biomarkers in hepatitis (HBV and HCV) mono-infected patients, the median sCD14 plasma levels were lower in patients with severe fibrosis (at 2.06 µg/mL) and in patients without fibrosis (at 1.74 µg/mL) [2]. Although we should be cautious when comparing these studies, sCD14 plasma levels seem to be lower in hepatitis mono-infected patients than in HIV/HCV co-infected patients [2], [3]. These differences in plasma levels might be result from the accelerated mechanism of fibrogenesis observed in HIV/HCV co-infection, which includes patients who are receiving a suppressive antiretroviral therapy [7], [8].

We found similar results to those of Marchetti et al. regarding the ability of inflammatory markers to predict the degree of liver inflammation and its progression to cirrhosis in HIV/HCV co-infected patients: these authors reported that a greater extent of fibrosis was not associated with higher sCD14 plasma levels [3]. This, however, contradicts Sandler et al.'s results, where sCD14 plasma levels were associated with cirrhosis and predicted progression to end-stage liver disease [4].

The difference between these studies is probably not related to differences in statistical power as (i) a similar number of patients were assessed in these studies; and (ii) sCD14 was associated with the stage of fibrosis, in univariate analyses, in both studies [4]. However, this difference may be because our study and Marchetti et al.'s study included HIV/HCV co-infected patients whereas Sandler et al.'s study focused on HBV/HCV mono-infected patients [3], [4]. HIV/HCV co-infected patients had higher levels of inflammatory biomarkers and sCD14 values because of the HIV co-infection, regardless of which stage of fibrosis. Thus, it seems to be more difficult to show an association between inflammation and progression to cirrhosis.

In our study, the level of the liver enzyme, AST, was the only factor independently associated with the extent of fibrosis. In HIV-infected patients, chronic elevation of AST and HCV infection have been previously described as independently associated with significant fibrosis [9]. When hepatitis is a mono-infection, AST has been correlated with sCD14 plasma levels, but was not associated with disease progression [9]. A previous study in HIV/HCV co-infected patients showed that heavy alcohol intake and increased AST levels were predictive of the severity of fibrosis [10].

In conclusion, in our series of HIV/HCV co-infected patients, sCD14 plasma level, the biomarker of monocyte activation, was not independently associated with the F3–F4 stage of liver fibrosis. Thus, we hypothesize that the higher levels of inflammation markers observed in HIV/HCV co-infected compared to HCV mono-infected patients may prevent such an association being observed in this population. Further studies that explore the possible roles of others biomarkers, for example the innate immune-activation marker, soluble CD163, may improve our understanding of the mechanisms of systemic immune activation.

## References

[1] Kalayjian RC, Machekano RN, Rizk N, Robbins GK, Gandhi RT, et al (2010) Pretreatment levels of soluble cellular receptors and interleukin-6 are associated with HIV disease progression in subjects treated with highly active antiretroviral therapy. J Infect Dis 201: 1796–1805. 20446847 10.1086/652750 PMC2873127

[2] Sandler NG, Wand H, Roque A, Law M, Nason MC, et al (2011) Plasma levels of soluble CD14 independently predict mortality in HIV infection. J Infect Dis 203: 780–790. 21252259 10.1093/infdis/jiq118 PMC3071127

[3] Marchetti G, Cozzi-Lepri A, Merlini E, Bellistrì GM, Castagna A, et al (2011) Microbial translocation predicts disease progression of HIV-infected antiretroviral-naive patients with high CD4+ cell count. AIDS 25: 1385–1394. 21505312 10.1097/QAD.0b013e3283471d10

[4] Sandler NG, Koh C, Roque A, Eccleston JL, Siegel RB, et al (2011) Host response to translocated microbial products predicts outcomes of patients with HBV or HCV infection. Gastroenterology 141: 1220–1230. 21726511 10.1053/j.gastro.2011.06.063 PMC3186837

[5] Rodger AJ, Fox Z, Lundgren JD, Kuller LH, Boesecke C, et al (2009) Activation and coagulation biomarkers are independent predictors of the development of opportunistic disease in patients with HIV infection. J Infect Dis 200: 973–983. 19678756 10.1086/605447 PMC2892757

[6] Boulware DR, Hullsiek KH, Puronen CE, Rupert A, Baker JV, et al (2011) Higher levels of CRP, D-dimer, IL-6, and hyaluronic acid before initiation of antiretroviral therapy (ART) are associated with increased risk of AIDS or death. J Infect Dis 203: 1637–1646. 21592994 10.1093/infdis/jir134 PMC3096784

[7] Thein HH, Yi Q, Dore GJ, Krahn MD (2008) Natural history of hepatitis C virus infection in HIV-infected individuals and the impact of HIV in the era of highly active antiretroviral therapy: a meta-analysis. AIDS 22: 1979–1991. 18784461 10.1097/QAD.0b013e32830e6d51

[8] Macías J, von Wichmann M, Rivero A, Tellez F, Merino D, et al (2010) Fast liver damage progression in HIV/HCV-co-infected patients in spite of effective HAART. [Abstract #659]. In: *Program and Abstract of the 17th Conference on Retroviruses and Opportunistic Infections;* 16–19 February 2010; San Francisco, USA.

[9] Vermehren J, Vermehren A, Mueller A, Carlebach A, Lutz T, et al (2012) Assessment of liver fibrosis and associated risk factors in HIV-infected individuals using transient elastography and serum biomarkers. BMC Gastroenterol 12: 27. 22453133 10.1186/1471-230X-12-27 PMC3361499

[10] Cartón JA, Collazos J, de la Fuente B, García-Alcalde ML, Suarez-Zarracina T, et al (2011) Factors associated with liver fibrosis in intravenous drug users coinfected with HIV and HCV. Antivir Ther 16: 27–35. 21311106 10.3851/IMP1708

